# Commentary: Exploratory research on therapeutic agents combined with early diagnostic biomarkers for colorectal cancer

**DOI:** 10.3389/fphar.2026.1820132

**Published:** 2026-05-08

**Authors:** Jingui Wan, Yuannan Lu, Ying Dai, Meixia Ye

**Affiliations:** Shenzhen Hospital (Futian) of Guangzhou University of Chinese Medicine, Shenzhen, Guangdong, China

**Keywords:** bioinformatics, colorectal cancer, drug repurposing, mechanistic validation, translational research

## Introduction

The relentless pursuit of novel biomarkers and therapeutic strategies for colorectal cancer (CRC) remains a cornerstone of oncology research, driven by the disease’s persistent global burden. The study by Liao and colleagues represents a commendable and timely effort in this arena, employing an integrated bioinformatics pipeline to bridge the gap between prognostic gene signatures and drug repurposing candidates (Liao et al., 2026). The authors harmonized data from TCGA and GEO cohorts to identify a panel of 15 survival-associated differentially expressed genes (DEGs). Their analysis converged on SB-225002 as a promising repositioned candidate. This finding was further supported by molecular docking studies and *in vitro* validation. This commentary aims to engage with the core intellectual contributions of this work. While we commend the integrative bioinformatics approach, we argue that the principal translational challenge lies in an unresolved mechanistic gap: the study correlates a prognostic gene signature with the drug candidate SB-225002 without establishing a causal biological link. Specifically, it remains unclear whether SB-225002s anticipated efficacy stems from its known role as a CXCR2 antagonist (affecting the tumor microenvironment) or from direct modulation of the signature genes (e.g., MELK, MCM2) as hypothesized based on molecular docking predictions. This gap between correlation and mechanism is the central focus of our critique, and we propose targeted experimental strategies to address it. While the original study frames its 15-gene signature in the context of early diagnosis, its analytical approach primarily establishes its value as a prognostic biomarker. A key translational question, and the focus of this commentary, is whether this prognostic signature can further serve as a predictive biomarker for the repurposed drug candidate SB-225002, and what mechanistic links underlie this potential association.

## Integration strengths and mechanistic verification gaps

The principal strength of this research lies in its systematic, multi-database integration strategy. However, this robust computational pipeline leads to a pivotal intellectual and translational quandary: the transition from identifying a prognostic gene signature to selecting a repurposed drug (SB-225002) is based on inverse correlation, leaving the underlying biological mechanism fundamentally unexplored. However, a pivotal intellectual question arises from the transition between biomarker discovery and therapeutic agent selection. SB-225002 is identified through its inverse correlation with the prognostic gene signature, yet its known primary mechanism is as a selective CXCR2 chemokine receptor antagonist (White et al., 1998). The CXCR2 axis remains an active therapeutic target in contemporary research, particularly for reprogramming the immunosuppressive tumor microenvironment in microsatellite stable colorectal cancer (Johnson, 2023). The study extensively details the binding affinity of SB-225002 to the protein products of the 15 genes via molecular docking, which generates hypotheses for potential off-target interactions. Nonetheless, the research does not experimentally bridge SB-225002s canonical anti-inflammatory pathway (CXCR2 antagonism) with the regulation of the identified oncogenic or tumor-suppressive genes (e.g., MELK, MCM2, ANGPTL1). This creates a conceptual gap: is the predicted efficacy of SB-225002 primarily mediated through its known CXCR2-axis modulation of the tumor microenvironment, or does it exert direct effects on these specific intracellular targets as indicated by the docking predictions? Notably, a recent study demonstrated that SB-225002 could induce cell death in CRC cells through a mechanism potentially independent of its canonical CXCR2 antagonism, underscoring the urgency to elucidate its precise molecular targets (Bazzichetto et al., 2025). To move from correlation to causation, future work must experimentally bridge this gap. This would require directly testing SB-225002s effect on the activity/expression of key signature genes (e.g., MELK) and dissecting the contribution of its canonical CXCR2 antagonism versus potential off-target effects.

## Translational context and biomarker panel utility

The study adeptly identifies a biomarker panel but leaves its practical clinical utility as a combined diagnostic or theranostic tool less explored. The construction of multi-gene prognostic signatures through integrated bioinformatics or machine learning approaches represents a prevalent and promising strategy in recent CRC research (Xun et al., 2024).​ The 15-gene signature includes both upregulated (e.g., MELK, NFE2L3) and downregulated (e.g., GREM2, CNN1) genes with complex, sometimes opposing, roles in cancer progression. While the authors provide a valuable literature synopsis for each gene, the collective biological coherence of this specific combination as a unified “early diagnostic” panel warrants deeper discussion. How might the concurrent measurement of these genes outperform existing single biomarkers or simpler panels? Furthermore, the connection between this specific gene signature and the susceptibility to SB-225002 treatment is postulated but not proven. A key, unresolved question is the predictive utility of this signature. The study posits a link between the signature and SB-225002 response, but does not test it. The critical next step is to determine whether the 15-gene signature can stratify models by their sensitivity to SB-225002. Successfully demonstrating such a correlation would fundamentally shift the panel’s value: from a prognostic indicator of general outcome to a predictive or theranostic tool capable of guiding therapy with a specific drug. This validation is essential for bridging the gap between computational discovery and clinical application in precision oncology, a critical step emphasized in contemporary methodologies for biomarker-guided drug repurposing (Wang et al., 2024). The schematic figure (presented below) elegantly summarizes the study’s robust integrative workflow and, crucially, highlights the key mechanistic gap and proposed future directions to strengthen the translational narrative.

**Figure:**
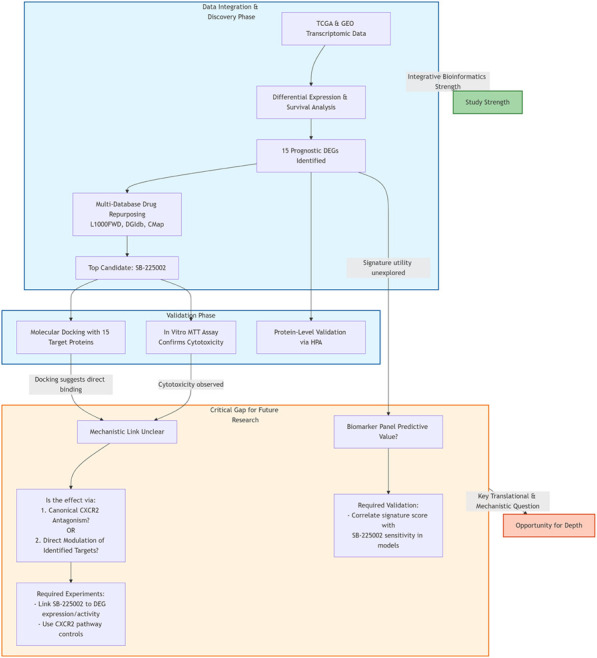


## Discussion

In conclusion, the work by Liao et al. delivers a valuable resource for the CRC research community. To address the mechanistic and translational gaps identified, two critical lines of experimental investigation are paramount: First, to delineate the mechanism of action of SB-225002 in CRC by concurrently assessing its impact on both the CXCR2 axis and the proteins of the prognostic signature (e.g., via target engagement and pathway dissection studies). Second, to evaluate the clinical utility of the 15-gene signature by testing its predictive power for SB-225002 response in stratified preclinical models. They have rigorously identified a prognostic gene signature and a plausible repurposed drug candidate. The critique presented here is not intended to diminish these achievements. Rather, it aims to frame them within the higher standards of mechanistic depth required for translational impact. The central opportunity for advancing this work lies in moving beyond correlation and affinity prediction to causal validation. Establishing whether SB-225002s efficacy in CRC is mediated through its expected CXCR2 pathway, the novel targets identified through docking studies, or a combination thereof, is essential. Similarly, testing the biomarker panel’s predictive power for drug response would bridge biomarker discovery with precision medicine. The field of drug repurposing benefits greatly from bioinformatics-driven studies like this one, yet their ultimate value is unlocked through subsequent experimental work that closes the loop between computational prediction and biological mechanism, as emphasized in recent discussions on translational bioinformatics (Xia et al., 2024). The presented framework is a solid foundation upon which such deeper, mechanistic investigations can be built, potentially accelerating the path toward novel combination strategies or patient stratification approaches in colorectal cancer.
